# Residual convolutional neural network for predicting response of transarterial chemoembolization in hepatocellular carcinoma from CT imaging

**DOI:** 10.1007/s00330-019-06318-1

**Published:** 2019-07-22

**Authors:** Jie Peng, Shuai Kang, Zhengyuan Ning, Hangxia Deng, Jingxian Shen, Yikai Xu, Jing Zhang, Wei Zhao, Xinling Li, Wuxing Gong, Jinhua Huang, Li Liu

**Affiliations:** 1 Hepatology Unit and Department of Infectious Diseases, Nanfang Hospital, Southern Medical University, Guangzhou, 510515 China; 2 Department of Oncology, The Second Affiliated Hospital of Guizhou Medical University, Kaili, China; 3 School of Biomedical Engineering, Southern Medical University, Guangzhou, China; 4 Department of Minimal Invasive Interventional Therapy, Sun Yat-Sen University Cancer Center, State Key Laboratory of Oncology in South China, Guangzhou, 510000 China; 5 Department of Radiology, Sun Yat-Sen University Cancer Center, State Key Laboratory of Oncology in South China, Guangzhou, China; 6 Department of Medical Imaging Center, Nanfang Hospital, Southern Medical University, Guangzhou, China; 7 Department of Interventional Radiology, Nanfang Hospital, Southern Medical University, Guangzhou, China; 8 Department of Oncology, Zhuhai Hospital Affiliated with Jinan University, Jinan University, Zhuhai, China

**Keywords:** Hepatocellular carcinoma, Artificial intelligence, Multidetector computed tomography, ROC curve

## Abstract

**Background:**

We attempted to train and validate a model of deep learning for the preoperative prediction of the response of patients with intermediate-stage hepatocellular carcinoma (HCC) undergoing transarterial chemoembolization (TACE).

**Method:**

All computed tomography (CT) images were acquired for 562 patients from the Nan Fang Hospital (NFH), 89 patients from Zhu Hai Hospital Affiliated with Jinan University (ZHHAJU), and 138 patients from the Sun Yat-sen University Cancer Center (SYUCC). We built a predictive model from the outputs using the transfer learning techniques of a residual convolutional neural network (ResNet50). The prediction accuracy for each patch was revaluated in two independent validation cohorts.

**Results:**

In the training set (NFH), the deep learning model had an accuracy of 84.3% and areas under curves (AUCs) of 0.97, 0.96, 0.95, and 0.96 for complete response (CR), partial response (PR), stable disease (SD), and progressive disease (PD), respectively. In the other two validation sets (ZHHAJU and SYUCC), the deep learning model had accuracies of 85.1% and 82.8% for CR, PR, SD, and PD. The ResNet50 model also had high AUCs for predicting the objective response of TACE therapy in patches and patients of three cohorts. Decision curve analysis (DCA) showed that the ResNet50 model had a high net benefit in the two validation cohorts.

**Conclusion:**

The deep learning model presented a good performance for predicting the response of TACE therapy and could help clinicians in better screening patients with HCC who can benefit from the interventional treatment.

**Key Points:**

*• Therapy response of TACE can be predicted by a deep learning model based on CT images.*

*• The probability value from a trained or validation deep learning model showed significant correlation with different therapy responses.*

*• Further improvement is necessary before clinical utilization.*

**Electronic supplementary material:**

The online version of this article (10.1007/s00330-019-06318-1) contains supplementary material, which is available to authorized users.

## Introduction

Hepatocellular carcinoma (HCC) ranks second as the major cause of cancer-related deaths globally and is the sixth most common cancer in the world. Its incidence has continuously increased in recent years, and approximately 850,200 new cases of HCC are annually diagnosed worldwide [1, 2]. Less than 30% of patients with HCC are eligible for potentially curative therapies, such as transplantation, resection, or ablation [3, 4]. For selected patients who are not suitable for such interventions, but have liver-confined disease, preserved liver function, and good performance status, transarterial chemoembolization (TACE) is recommended according to international guidelines [5–9].

Although repeated TACE procedures are often needed, the initial response effectively predicts the overall survival (OS) because the best response cannot always be achieved after one session of TACE, especially in large tumors. Moreover, the achievement of a treatment response at an early time point is the robust predictor for favorable outcomes [10]. The texture analysis based on contrast-enhanced magnetic resonance imaging (MRI) before TACE may act as imaging biomarkers to predict an early response from patients with HCC. The highest accuracy for complete response (CR) group and the non-complete response (NCR) was 0.76 [11]. A pretherapeutic dynamic CT texture analysis can also be valuable in predicting the therapy response of HCC to TACE. Higher arterial enhancement and GLCM (gray-level co-occurrence matrix) moments, lower homogeneity, and smaller tumor size are significant predictors of complete response (CR) after TACE [12]. However, based on the traditional statistics and machine learning, the accuracy of this method is limited. On the condition of optimal cutoff values for predicting a CR to TACE in the receiver operating characteristic (ROC) curves, the highest AUC of texture parameters was 0.72. Furthermore, most studies focused on the two classifications (CR or NCR) and the prediction of four classifications (CR, PR, SD, and PD) using CT images is unclear. Therefore, a more effective model to accurately identify patients who would have an initial response after TACE therapy is urgently needed to facilitate individualized treatment strategies.

Deep learning has recently gained attention as a technique for realizing Artificial intelligence (AI) [13–15]. Several types of deep-stacked artificial neural networks, such as convolutional neural networks (CNNs) and recurrent neural networks (RNNs), have been proposed and judiciously used in various fields. Deep CNNs are especially recognized as demonstrating high performance for image recognition tasks [16, 17]. Some initial successes in applying deep learning to the assessment of radiological images have been witnessed [18–21]. A study used a deep learning algorithm to non-invasively predict the IDH (Isocitrate Dehydrogenase) status within a multi-institutional dataset of low- and high-grade gliomas [22]. Deep learning also shows the potential to stage liver fibrosis based on radiological images [23]. However, there is embarrassed that performing deep learning often faces a shortage of medical data, especially in radiological images of patients undergoing treatment. Transfer learning, which is a feasible deep learning technique for addressing a lack of image data, has been proven a highly effective technique, particularly in the case of limited medical images [24, 25]. The models have been used to distinguish the features of the medical images in a much faster manner and with significantly fewer training medical images [13].

In this study, based on the CT images from three independent centers, we aimed to investigate a deep learning algorithm to precisely and non-invasively evaluate the different therapy response in HCC patients before the TACE treatment.

## Materials and methods

### Patients

Our retrospective study had been approved by the institutional review board and Ethical Committee (NFEC-201208-K3). This study included patients in Nan Fang Hospital (NFH), Zhu Hai Hospital Affiliated with Jinan University (ZHHAJU), and Sun Yat-sen University Cancer Center (SYUCC), who matched the following criteria for selection: (a) radiologically or pathologically proven HCC; (b) received initial treatment of TACE; (c) availability of hepatic-arterial CT imaging within 7 days before treatment; (d) availability of hepatic-arterial CT imaging within 30 days after treatment; and (e) patients with Barcelona Clinic Liver Cancer (BCLC) stage B. The exclusion criteria were as follows: (a) previous treatments, including loco-regional or whole-body therapies, such as liver transplantation, radiotherapy, radiofrequency ablation, or sorafenib treatment, and (b) other malignant liver tumors. Electronic medical records were used to collect the pretreatment clinical characteristics of patients. Supplemental Figure 1 shows the recruitment pathways for patients in training and two validation cohorts. Based on the radiology evaluation in patients after the first TACE therapy, the different responses on hepatic-arterial CT images were determined by modified RECIST (mRECIST 1.1), including complete response (CR), partial response (PR), stable disease (SD), and progressive disease (PD). We defined the objective response as CR+PR and the non-response as SD+PD.

### CT acquisition and region-of-interest segmentation

Contrast-enhanced computed tomography (CECT) was performed at three hospitals as previously described [26]. Supplemental Table S1 presents the scan characteristics for the three centers. All CT images were downloaded using a picture archiving and communication system (PACS; Nan Fang Hospital, Zhu Hai Hospital Affiliated with Jinan University, and Sun Yat-sen University Cancer Center Network Center, China). The CT images of all patients were input into the ITK-SNAP software (version 3.6). The tumor regions of interest (ROIs) were analyzed by three senior radiologists who had 14-year experience (reader 1, Jing Zhang), 17-year experience (reader 2, Jingxian Shen), and 23-year experience (reader 3, Yikai Xu). The ROIs of CT images from the training cohort (NFH) and two validation cohorts (ZHHAJU and SYUCC) were manually segmented by reader 1 and reader 2, respectively. Then, reader 3 confirmed each ROI and saved as the main file (CT image) and segmentation file (mask image) in the ITK-SNAP software. All radiologists were specifically blinded to the therapy outcome of the patients from three cohorts.

### Image preprocessing

The window width and level were transformed into the original one. All CT images were reconstructed using a medium sharp reconstruction algorithm with a thickness of 1 mm. Subsequently, the intensity values of the image were mapped to [0, 1]. This target of the deep learning algorithm is the classified labels of CR, PR, SD, and PD. We saved one CT image and mask of ROI from the largest tumor area for each patient and then saved the other two CT images and two corresponding masks of ROIs from the nearest sequences. Using an in-house algorithm, we extracted an average of approximately three patches with a resize of 224 × 224 × 3 for each patient in the training and two validation cohorts, respectively. Notably, each patch for the training and validation of the network was entirely included in the ROIs of the CT images. Finally, 1687 patches were extracted from 562 patients for training (NFH); 268 patches were extracted from 89 patients for validation 1 (ZHHAJU); and 406 patches were extracted from 138 patients for validation 2 (SYUCC). Data augmentation techniques were introduced before the training procedure considering the potential bias caused by the unbalanced data and big data requirement of deep learning [27]. Specifically, in our study, the patches were equally and randomly distributed across each class by data augmentation, including level flip, vertical flip, level and vertical flip, 90° rotation, and − 90° rotation. Using this method, we built a “new” training data and this augmentation was only performed on the NFH cohort, not on the two external validation cohorts. In order to minimize memory usage, we used data augmentation only in real time.

### Transfer learning of the residual neural network

**Fig. 1:**
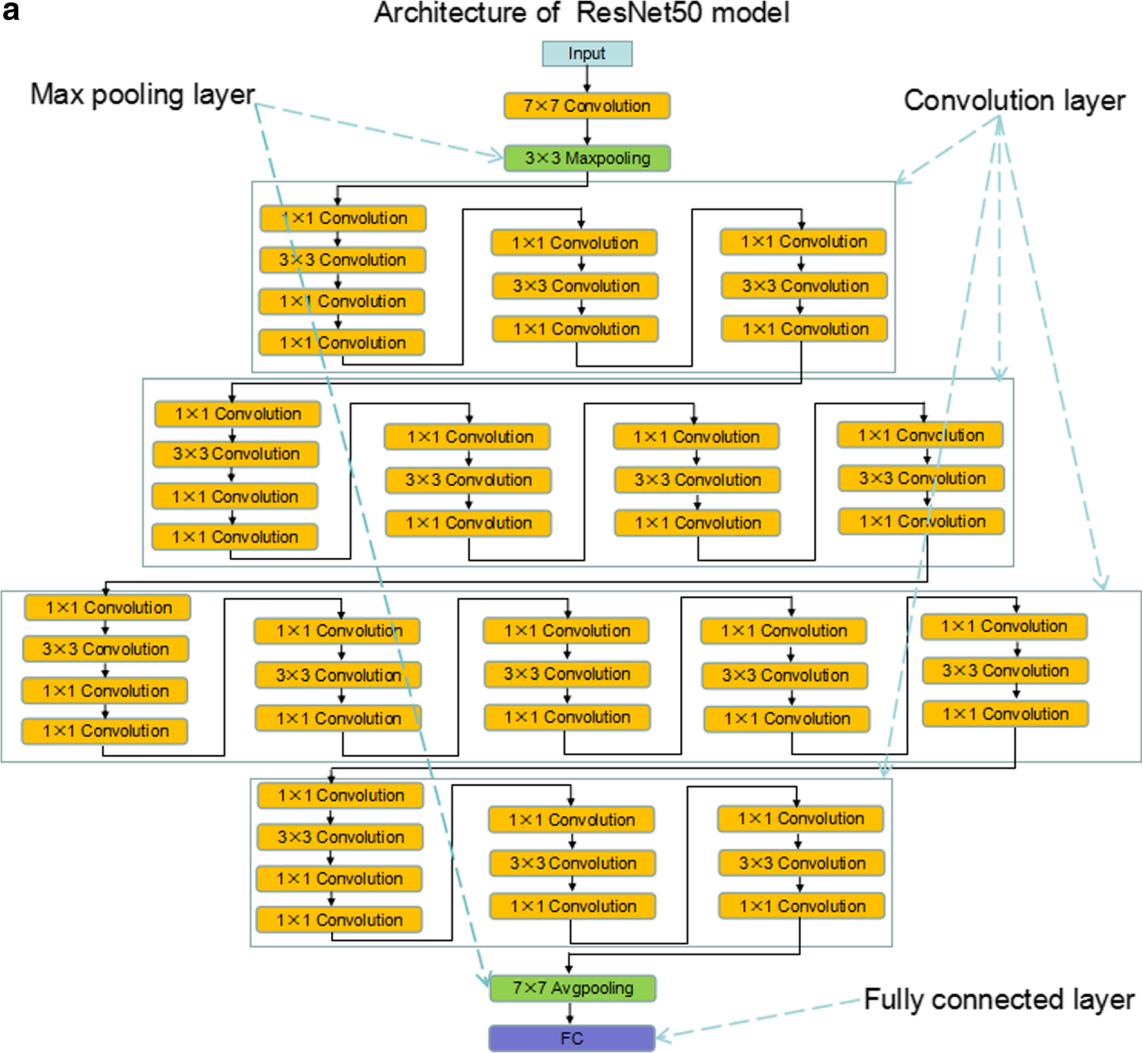

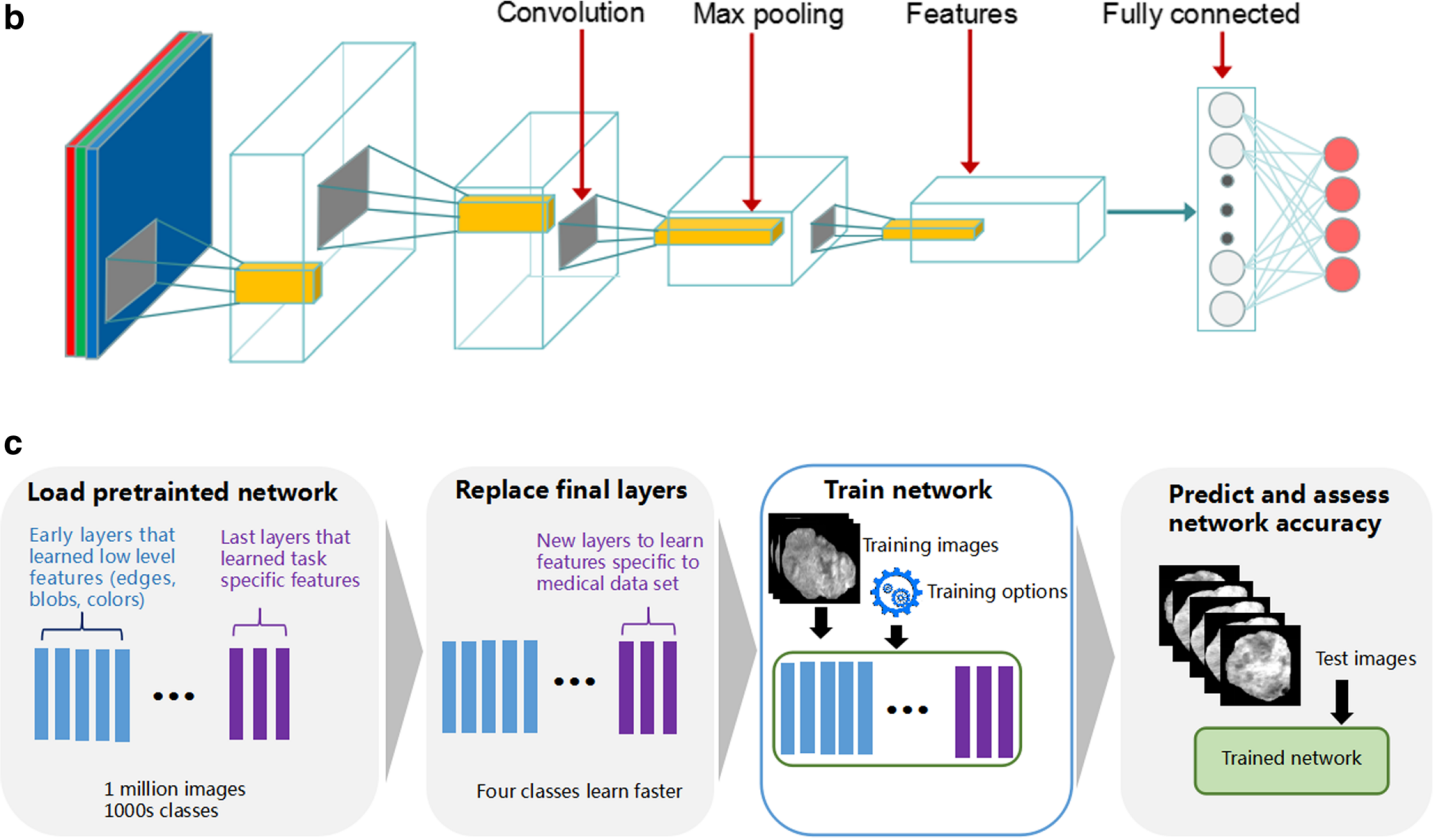
The architecture of ResNet50 and deep learning model flowchart. **a**, **b** Architecture of ResNet50 is shown and includes convolution layers, max pooling layers, and a fully connected layer. **c** A ResNet50 model was pretrained on a million images from the ImageNet database and can classify images into 1000 object categories. Based on this new dataset of CT images, a transfer learning model was adapted to significantly shorten the training time and improve the accuracy. The earlier connected layers were frozen (1 to 174), and the final connected layers were replaced (175 to 177). Finally, this model was transferred to a novel network. All patches were augmented in the proposed approach after the ROI patches were determined from the CT images. A transfer learning 50-layer residual convolutional neural network was used to predict the response to TACE therapy

ResNet is a representative deep convolutional neural network integrated with images, auto-encoding, and classification. It won the 2015 ImageNet Large Scale Visual Recognition Challenge (ILSVRC) by using feature transmission to prevent gradient vanishing, such that a much deeper network than those used previously could be effectively trained [28]. Motivated by a previous study that a deeper network is potentially more powerful than shallow networks, our residual network was derived from a 50-layer residual network architecture and has 177 layers in total, showing detailed information in the Supplementary Information (ResNet50). ResNet50 has been trained on a subset of the ImageNet database (http://www.image-net.org) and can classify images into 1000 object categories (e.g., keyboard, mouse, pencil, and many animals). The architecture of ResNet50 and flowchart of deep learning for CT images were shown in Fig. 1a–c. We froze the weights of earlier layers (1 to 174) in the pretrained network. The trained network does not update the parameters of the frozen layers. Freezing the weights of many initial layers can significantly speed up network training and prevent over-fitting to the new medical dataset.

A series of blocks consisting of three convolutional layers (fc1000, fc1000_softmax, and classification layers_fc1000) were replaced by new layers (fc4, fc4_softmax, and classification layers_fc4) to extract deep residual features and transmit features from the front layer to the latter one. At the end of the network, a full-connection layer was used to perform classification. During training, the weights were optimized via the stochastic gradient descent (SGD) optimization algorithm with a mini-batch size 64 [29]. After fine-tuning parameters of deep learning, the learning rate and the number of max epochs were set to 0.0001 and 54, respectively, to ensure covering of the entire data for efficient training. The loss function was identified as binary cross-entropy. We used the sigmoid function to compute the probability before the output layer. The performance of deep learning model was estimated by AUC and accuracy ($Accuracy=\frac{1}{n}\sum 1\left( yi= ti\right)$). The patches from Nan Fang Hospital (NFH) were trained via pretrained ResNet50. Based on the trained model, patches from Zhu Hai Hospital Affiliated with Jinan University (ZHHAJU) and Sun Yat-sen University Cancer Center (SYUCC) were used to be validated, respectively.

### Implementation details

Our implementation was based on the Deep Learning Toolbox™ Model for the ResNet50 Network in MATLAB (version 2018a; MathWorks). Our training experiments were performed in a Linux environment on a machine with the following specifications: CPU Intel Xeon Processor E5-2640V3 at 2.60 GHz, GPU NVIDIA Pascal Titan X, and 128-GB RAM.

### Statistical analysis

Statistical analyses were performed with R statistical software version 3.5.0 (R Core Team, 2018), GraphPad prism 7.0, and MATLAB 2018a. The receiver operating characteristic (ROC) curves were plotted with the “pROC” package. A confidence interval (CI) of 95% for AUC was calculated using each dataset (1000 bootstrap). The confusion matrices were plotted with MATLAB 2018a in three different cohorts to calculate the accuracies of estimating the response of TACE therapy. Decision curve analysis (DCA) was performed using the “dca.R” package. The Mann–Whitney U test was analyzed by R statistical software. Two-sided *p* values < 0.05 were considered significant.

## Results

### Clinical characteristics of patients

Five hundred sixty-two patients with HCC were finally included in the training cohort (NFH), and 89 and 138 patients were allocated to the independent validation cohorts 1 (ZHHAJU) and 2 (SYUCC), respectively. Table 1 summarizes the baseline clinical characteristics of the training and two validation cohorts. In the training cohort, validation cohort 1, and validation cohort 2, the age of 168 (29.90%), 28 (31.46%), and 38 (27.54) patients was more than 60 years, respectively. Sixty (10.68%), 14 (15.73%), and 15 (10.87%) patients were females in the training cohort, validation cohort 1, and cohort 2, respectively. In the training cohort, the patients for CR, PR, SD, and PD were 83 (14.77%), 151 (26.87%), 222 (39.50%), and 106 (18.86%), respectively. In validation cohorts 1 and 2, the patients for CR, PR, SD, and PD were 13 (14.61%) and 21 (15.22%), 24 (26.97%) and 37 (26.81%), 35 (39.33%) and 54 (39.13%), and 17 (19.09%) and 26 (18.84%), respectively. No significant differences were observed between the three cohorts in the clinical database.

**Table 1:** Participant characteristics in the training and validation cohorts

Characteristic	Training cohort (*n* = 562)	Validation cohort 1 (*n* = 89)	Validation cohort 2 (*n* = 138)
Age (years)			
≤ 60	394 (70.10%)	61 (68.54%)	100 (72.46%)
> 60	168 (29.90%)	28 (31.46%)	38 (27.54%)
Sex			
Male	502 (89.32%)	75 (84.27%)	123 (89.13%)
Female	60 (10.68%)	14 (15.73%)	15 (10.87%)
HBsAg status			
Positive	514 (91.46%)	80 (96.67%)	125 (90.57%)
Negative	48 (8.54%)	9 (3.33%)	13 (9.43%)
Child–Pugh classification			
A	451 (80.25%)	74 (83.15%)	111 (80.43%)
B	111 (19.75%)	15 (16.85%)	28 (19.57%)
ALT (U*/*mL)			
≤ 40	240 (42.70%)	45 (50.56%)	69 (50.00%)
> 40	322 (57.30%)	44 (49.44%)	69 (50.00%)
AST (U*/*mL)			
≤ 40	147 (26.16%)	27 (30.34%)	37 (26.81%)
> 40	415 (73.84%)	62 (69.66%)	101 (73.19%)
AFP (ng/mL)			
≤ 20	322 (57.30%)	49 (55.06%)	83 (60.14%)
> 20	240 (42.70%)	40 (44.94%)	55 (39.86%)
Hepatocirrhosis status			
Present	294 (52.31%)	40 (44.94%)	70 (50.73%)
Absent	268 (47.69%)	49 (55.06%)	68 (49.27%)
Tumor size (cm)			
≤ 5	83 (14.77%)	12 (13.48%)	22 (15.94%)
> 5, ≤ 10	245 (43.59%)	37 (41.57%)	52 (37.68%)
> 10	234 (41.64%)	40 (44.95%)	64 (46.38%)
Tumor numbers			
≤ 3	463 (82.38%)	77 (86.51%)	114 (82.61%)
> 3	99 (17.62%)	12 (13.49%)	24 (17.39%)
Response to therapy			
CR	83 (14.77%)	13 (14.61%)	21 (15.22%)
PR	151 (26.87%)	24 (26.97%)	37 (26.81%)
SD	222 (39.50%)	35 (39.33%)	54 (39.13%)
PD	106 (18.86%)	17 (19.09%)	26 (18.84%)

*HBsAg*, hepatitis B surface antigen; *ALT*, alanine aminotransferase; *AST*, aspartate aminotransferase; *AFP*, alpha-fetoprotein; *CR*, complete response; *PR*, partial response; *SD*, stable disease; *PD*, progressive disease

### Training and validation of the deep learning model in multi-class classification

**Fig. 2:**
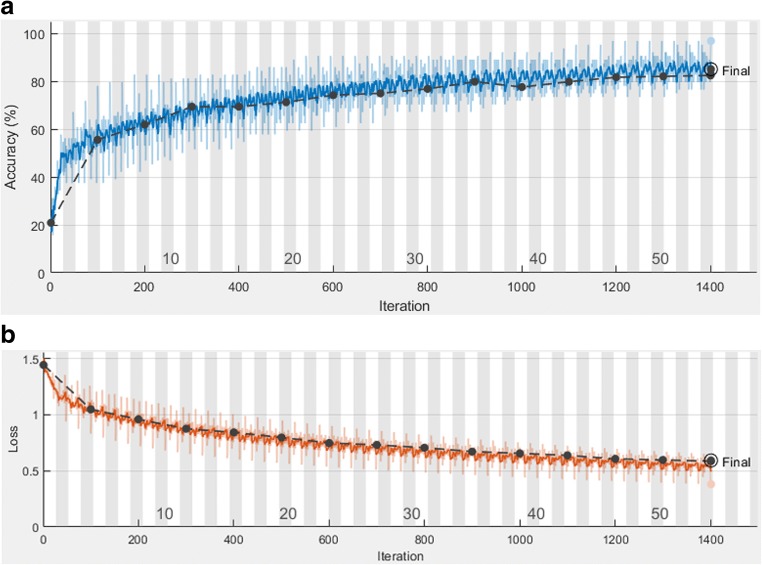
Training and validation processes of the deep learning model based on the CT images. Accuracy (**a**) and cross-entropy loss (**b**) were plotted against the training step during the length of the training of the four-class classifier over the course of 54 steps. The red and black lines represent the training and validation processes, respectively. The cross-entropy loss was close to 0.5, while the final validation accuracy was 85.07%

**Fig. 3:**
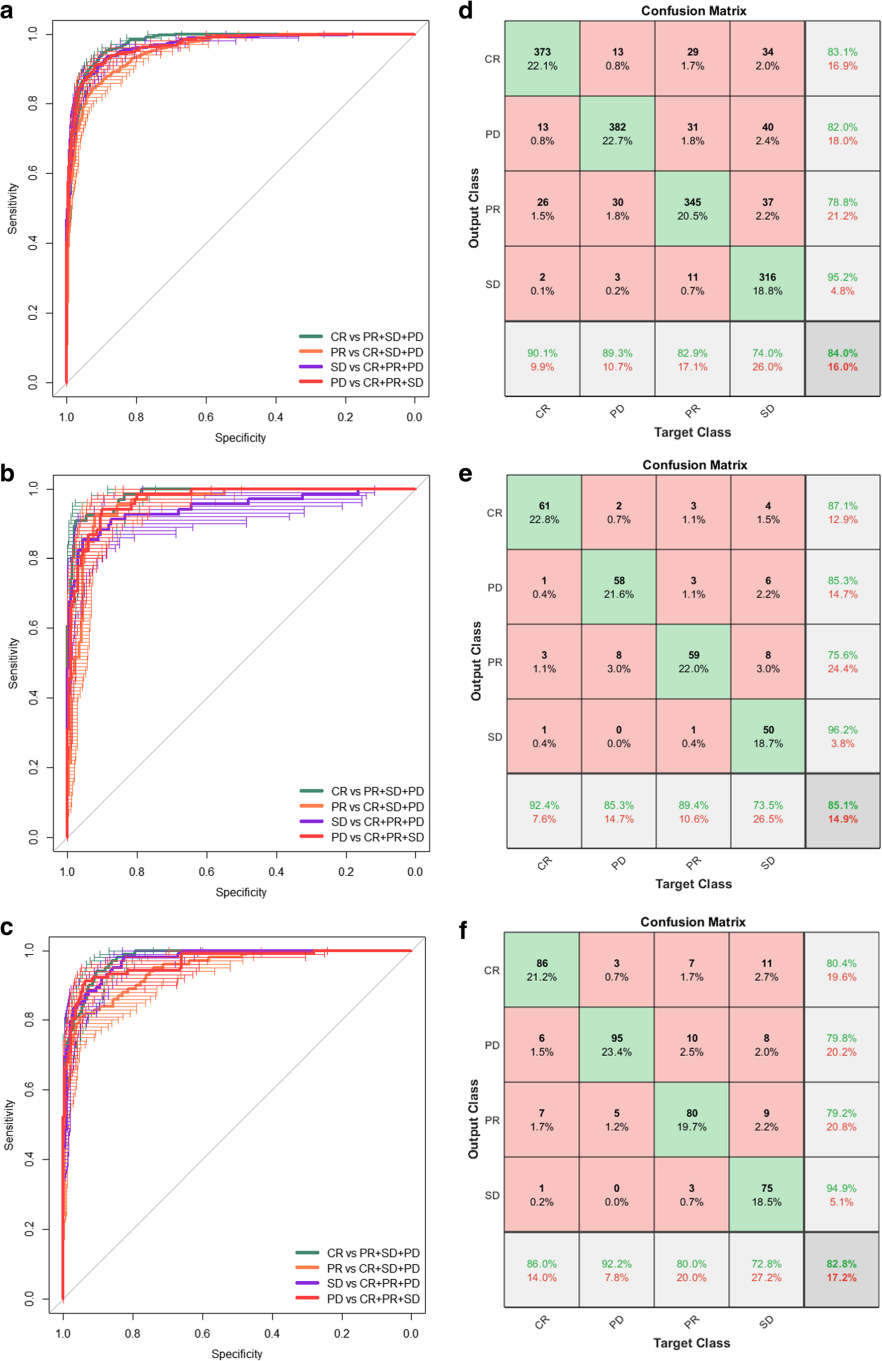
ROC curve and confusion matrix for predicting the response to TACE therapy. **a** Each patient for the assessment of the response to TACE therapy is shown via the ROC curve. In the training set, the deep learning model had an AUC of 0.97, 0.96, 0.95, and 0.96 for CR, PR, SD, and PD, respectively. **b** In validation 1 set, the deep learning model had an AUC of 0.98, 0.96, 0.95, and 0.94 for CR, PR, SD, and PD, respectively. **c** In validation 2 set, the deep learning model had an AUC of 0.97, 0.96, 0.95, and 0.95 for CR, PR, SD, and PD, respectively. **d**–**f** The model exhibited accuracies of 84.3%, 85.1%, and 82.8% in the three cohorts (NFH, ZHHAJU, and SYUCC), respectively

All the patches (*n* = 1687) were augmented and trained in the training cohort via the residual convolutional neural network (ResNet50) to increase the robustness of the model. Figure 2a shows that the accuracy was above 80% and the cross-entropy loss was close to 0.5 after 54 epochs training (1401 iterations) and 71 min 38 s time (Fig. 2b). The resulting model had an AUC of 0.97 (0.97–0.98), 0.96 (0.96–0.97), 0.95 (0.94–0.96), and 0.96 (0.96–0.97) for CR, PR, SD, and PD, respectively (Fig. 3a). We then tested the patches (*n* = 268) in validation cohort 1. An AUC of 0.98 (0.97–0.99), 0.96 (0.95–0.98), 0.95 (0.93–0.98), and 0.94 (0.90–0.98) for CR, PR, SD, and PD was observed (Fig. 3b). In validation cohort 2, the AUCs of predicting CR, PR, SD, and PD in TACE treatment were 0.97 (0.96–0.98), 0.96 (0.94–0.98), 0.94 (0.92–0.97), and 0.97 (0.95–0.98), respectively (Fig. 3c). The deep learning model indicated good discrimination of the therapy response using the ROI patches in the two cohorts.

**Fig. 4:**
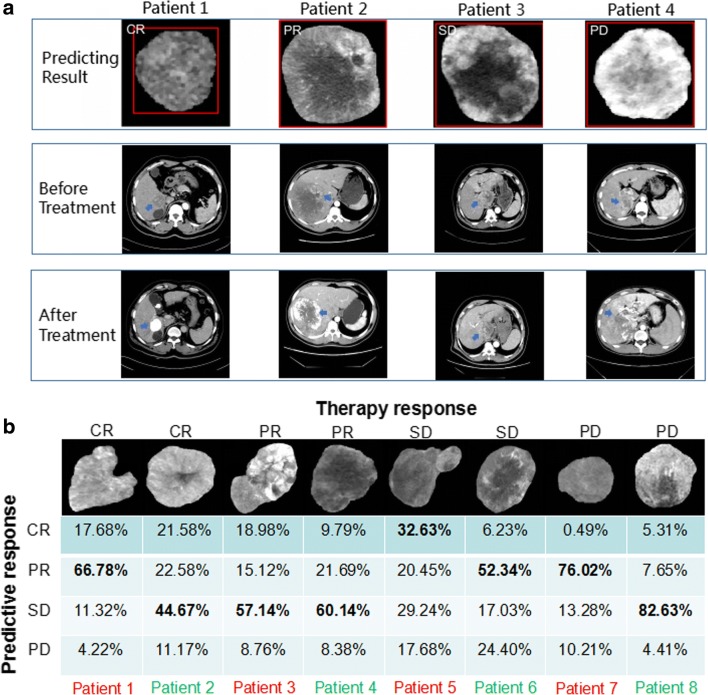
Examples of correctly predicted and misclassified patches by the ResNet50. **a** Horizontal cross-section CT images through four patients of validation cohort 2 with HCC before and after 1 month of TACE therapy. **b** Eight patches showed the misclassifications of four therapy responses in validation cohorts 1 and 2, respectively. The output of the deep learning model is presented below each patch. Red and green colors represent the ROI images from validation cohorts 1 and 2, respectively

We next focused on the predictive accuracy of the deep learning model in each patch by confusion matrix. The training cohort exhibited an average accuracy of 84.0% and a low error of 16% of predicting CR, PR, SD, and PD in TACE therapy (Fig. 3d). The independent validation cohorts 1 and 2 showed an average accuracies of 85.1% and 82.8% and low errors of 14.9% and 17.2%, respectively (Fig. 3e, f). We further chose four typical patches of different therapy responses from validation cohort 2 and displayed the CT images of the maximum cross-sectional diameter of the liver tumor, including pretreatment and post-treatment of the hepatic-arterial phase images (Fig. 4a). As shown, the deep learning model performed well in the classification of predicting CR, PR, SD, and PD of TACE treatment. There were some difficult cases of these patterns that were misclassified in the ResNet50. We displayed eight misclassified images of ROI from validation cohorts 1 and 2 in Fig. 4b. For example, CR could be incorrectly predicted as SD (44.67%) in patient 2, and PD also could be incorrectly predicted as PR (76.02%) in patient 7.

### Evaluation on performance of two-class classification via trained ResNet50 model

**Fig. 5:**
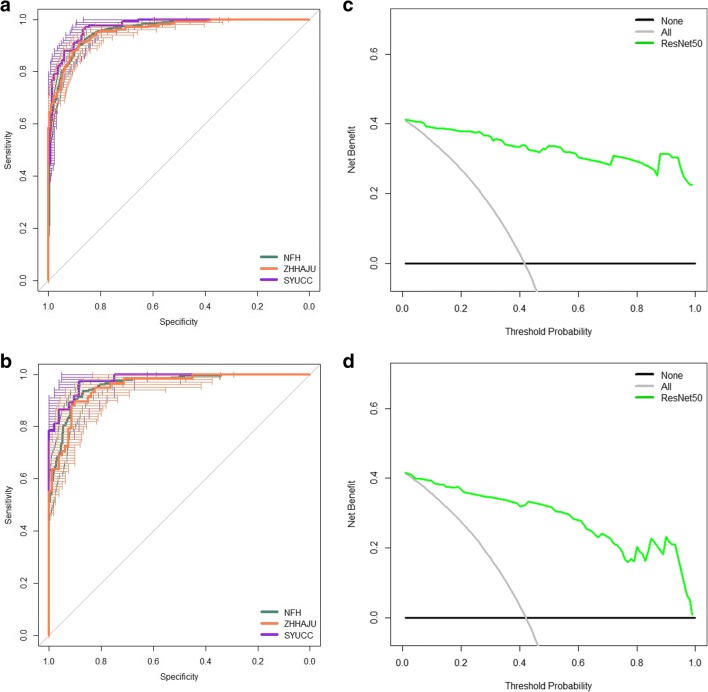
ROC curve and DCA curve for estimating the objective response to TACE therapy. **a** In the NFH, ZHHAJU, and SYUCC cohorts, the deep learning model had an AUC of 0.95, 0.96, and 0.97 for predicting therapy response via patches, respectively. **b** Based on the predictive probability, the model presented an AUC of 0.95, 0.96, and 0.97 for predicting therapy response in all patients from NFH, ZHHAJU, and SYUCC cohorts, respectively. In the ZHHAJU (**c**) and SYUCC (**d**) cohorts, the DCA indicated that when the threshold probability was above 2% and 4%, THE use of the deep learning model for predicting TACE response would gain more benefit than the “treat-all” patients or “treat-none” schemes

Multiple 2D arrays were output by the convolutional layers of a ResNet50 model. To further estimate the classification performance of our deep network trained on four-category learning, we have considered a clinically important task, including two-class classification of objective response (CR or PR) and non-response (SD or PD). On the task of two-class classification, we have merged the output prediction probability by the trained deep learning model for four-category classification, for adding CR and PR (i.e., *y*^response^ = *y*^CR^ + *y*^PR^). The probability value of therapy response (objective response) was significantly increased in the response HCC patches versus the non-response HCC patches in Supplemental Figure 2A–C (each cohort, *p* < 0.0001). Throughout this method, the model achieved an AUC of 0.95 (0.95–0.96), 0.96 (0.94–0.97), and 0.97 (0.96–0.98) in the patches from NFH, ZHHAJU, and SYUCC cohorts (Fig. 5a). The probability value of therapy response was calculated bythe average probability values of all patches in each patient of three cohorts. We found the ResNet50 model presented high AUC in the patients of three cohorts (Fig. 5b). To analyze the clinical use of model based on deep learning, the decision curve analysis (DCA) for the predictor of ResNet50 was performed in this study. When the threshold probabilities are ~ 2% and 4%, the model shows stronger benefit in comparison with the treat-all or treat-none strategy in the ZHHAJU (Fig. 5c) and SYUCC (Fig. 5d) cohorts. This similar performance of DCA also has been displayed in the training cohort (Supplemental Figure 3).

## Discussion

In this study, we demonstrated a novel application of deep learning to predict the response of TACE therapy in a three-institution dataset of HCC. As far as we know, this is the first time that the deep learning model based on radiological images is used to predict the four responses of interventional treatment in liver cancer. This algorithm may facilitate deep learning techniques for the medical field of precise therapy oncology. Based on the pretreatment ROI images of patients with HCC, this utility model of deep learning is a potential method for predicting the response of TACE therapy.

According to the BCLC stage system, TACE is recommended for patients with HCC with BCLC stage B [6, 30–32]. In patients with stage C, TACE therapy is also a frequent and important application treatment, especially in comprehensive treatment [33–35]. Recent studies revealed that the therapy response at first chemoembolization is a good predictor for the favorable outcome in hepatocellular carcinoma [10, 36]. However, no report was associated with the prediction response of TACE therapy in the field of hepatocellular carcinoma via deep learning of CT images. Previous studies showed that different clinical risk factors (e.g., tumor size) perform well in prediction [37–39], but the precise estimation of four therapy responses remains challenging in clinical settings and difficult to implement. A new method involving a radiomics approach based on radiological images (e.g., CT, MR, and PET-CT) is also currently being applied in various tumors. It extracts radiographic features from conventional images and includes the features of tumor shape, texture, intensity, and wavelet transform characteristics [40–46]. However, numerous pre-engineered features are artificial design features. This may lead to poor reproducibility and nonredundant radiomics features (RFs) for CT images because of the variable scan parameters of different types of imaging equipment [47]. The application of radiomics also relies on traditional machine learning techniques. Unlike the above method, the algorithm of deep learning can directly learn predictive features from the images and potentially greatly increase the robust accuracy in these radiological images [48, 49].

Previous studies mostly focused on deep learning based on the small segmentation patches of each ROI image to enhance the size of the sample and frequently showed a significant AUC [50–52]. However, the scenario of predicting the labels of entire ROI images was often ignored. Therefore, we used an algorithm of transfer learning to evaluate the treatment response via the whole CT-ROI patches. Comparing with previous study, high AUCs of predicting CR, PR, SD, and PD in the therapy response of TACE were observed among the three cohorts [12]. This result indicated that our transfer learning model performed well in predicting different therapy responses using CT images from three independent centers. The result of the confusion matrix presented significantly high accuracies of prediction in the NFH, ZHHAJU, and SYUCC cohorts and was distinct from previous report [11]. Interestingly, we found the accuracy for the training cohort was lower than for the validation cohort 1 (84.3% vs. 85.1%). We speculated the phenomenon was correlated with a small sample size of patients in validation cohort 1. Increasing number of patients would potentially reduce the validation accuracy. Misclassified CR patches by the deep learning model were more observed in PR patches than in SD and PD patches in the training cohort (1.5%) and validation cohorts 1 (1.7%) and 2 (1.1%). Meanwhile, misclassified PD patches were more frequently found in PR patches than in SD and CR patches. The precision probability of preoperatively predicting the four therapy responses (i.e., CR, PR, SD, and PD) via each ROI patch was calculated and found useful in individualized clinical treatment. We further investigate the prediction of objective response (response or non- response) in patches or patients and also found high accuracies in the three cohorts. This finding demonstrated that the deep learning model based on CT images may help doctors recognize patients who would acquire well or poor initial response of TACE therapy.

However, our study has several limitations. First, the sample size of patients with HCC was relatively small, and this was a retrospective research. A much larger database of the prospective study would be collected from more centers in the future. Second, we trained and validated all the patches of the 2D CT images from three medical centers. Because of 3D patches’ potential of having more context information, we speculated the 3D CT patches had an accuracy higher and a better model quality than that of the 2D CT patches. The 3D CT patches would be investigated in the next step. Third, the correlation between the biological processes (e.g., differential gene expression and pathway) and the prediction results of deep learning networks in HCC was unknown and should be analyzed in the future. Fourth, the ROIs were drawn manually in our study. Lesions selected from different abdominal radiologists might have various differences, impacting on disease classification. We would use the combination of the algorithm for HCC segmentation and ResNet50 model to automatically predict the outcome of TACE therapy in the following study.

In summary, the deep learning model based on CT images would potentially serve as a new tool for predicting the therapy response of patients undergoing TACE treatment. Our method using transfer learning for predictive classification of radiological images may also be used to determine more precise clinical treatments in other malignant tumors.

## Electronic supplementary material

ESM 1 (DOCX 3103 kb)

## Abbreviations

AI: Artificial intelligence
AUCs: Areas under the curve
BCLC: Barcelona Clinic Liver Cancer
CECT: Contrast-enhanced computed tomography
CI: Confidence interval
CNNs: Convolutional neural networks
CR: Complete response
DCA: Decision curve analysis
GLCM: Gray-level co-occurrence matrix
HCC: Hepatocellular carcinoma
IDH: Isocitrate dehydrogenase
ILSVRC: ImageNet Large Scale Visual Recognition Challenge
MRI: Magnetic resonance imaging
NCR: Non-complete response
NFH: Nan Fang Hospital
OS: Overall survival
PACS: Picture archiving and communication system
PD: Progressive disease
PR: Partial response
RFs: Radiomics features
ROC: Receiver operating characteristic
ROIs: Regions of interest
RNNs: Recurrent neural networks
SD: Stable disease
SGD: Stochastic gradient descent
SYUCC: Sun Yat-sen University Cancer Center
TACE: Transarterial chemoembolization
ZHHAJU: Zhu Hai Hospital Affiliated with Jinan University
