# Supplementary material for: Residual convolutional neural network for predicting response of transarterial chemoembolization in hepatocellular carcinoma from CT imaging
Source: Eur Radiol. 2019 Jul 22;30(1):413–24. doi: 10.1007/s00330-019-06318-1 (PMC6890698; doi:10.1007/s00330-019-06318-1)
Supplement: Supplementary file 1 — (DOCX 3103 kb) [file 330_2019_6318_MOESM1_ESM.docx]

**Supplementary Information**

1. **Computed tomography image acquisition parameters**

All patients underwent pre-treatment multi-detector row CT (MDCT) scans (SOMATOM and 256-iCT). The acquisition parameters were as follows:

| **Table 1. Scanning parameters for each scanner** | | |
| --- | --- | --- |
| Parameter | SOMATOM (ZHHAJU) | 256-iCT (NFH and SYUCC) |
| Tube voltage (kVp) | 120 | 120 |
| Tube current (mA) | Auto | Auto |
| Detector collimation (mm) | 64×0.6 | 128×0.625 |
| Field of view (mm) | 250 | 250–400 |
| Pixel size | 512×512 | 512×512 |
| Rotation time (s) | 0.5 | 0.4 |
| Slice interval (mm) | 0 | 0 |
| Slice thickness (mm) | 5 | 5 |

**
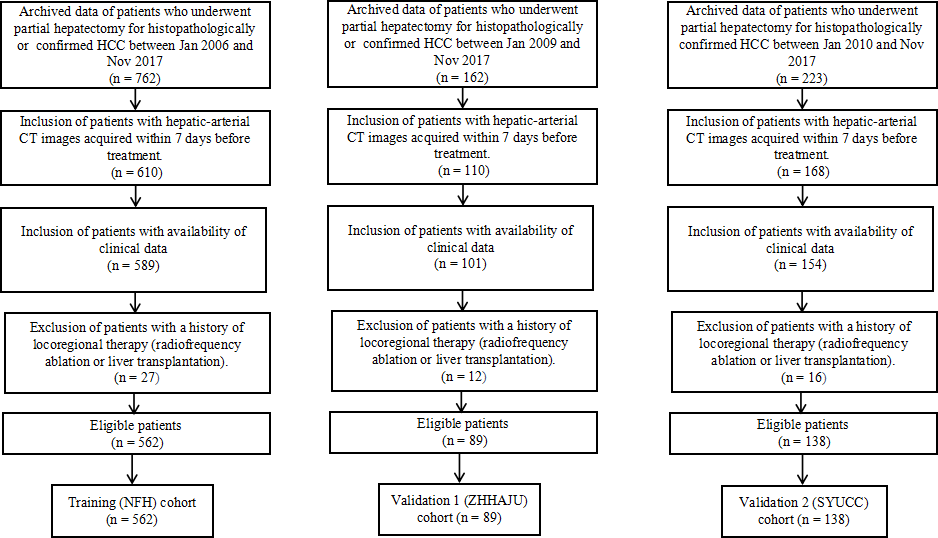
**

**Supplemental Figure 1.** Flowchart shows the process of recruitment pathways for patients in training and two validation cohorts.


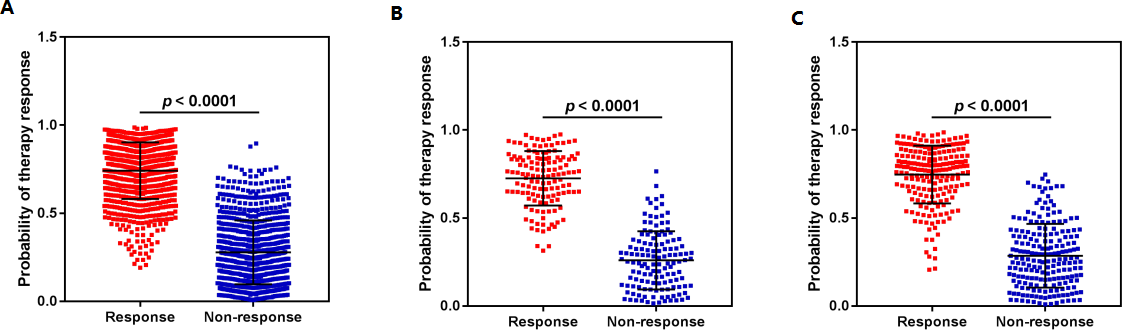


**Supplemental Figure 2.** The probability value of therapy response was significantly high in the response HCC patches versus the non-response HCC patches (each cohorts *P* < 0.0001). A, NFH cohort; B, ZHHAJU; C, SYUCC.


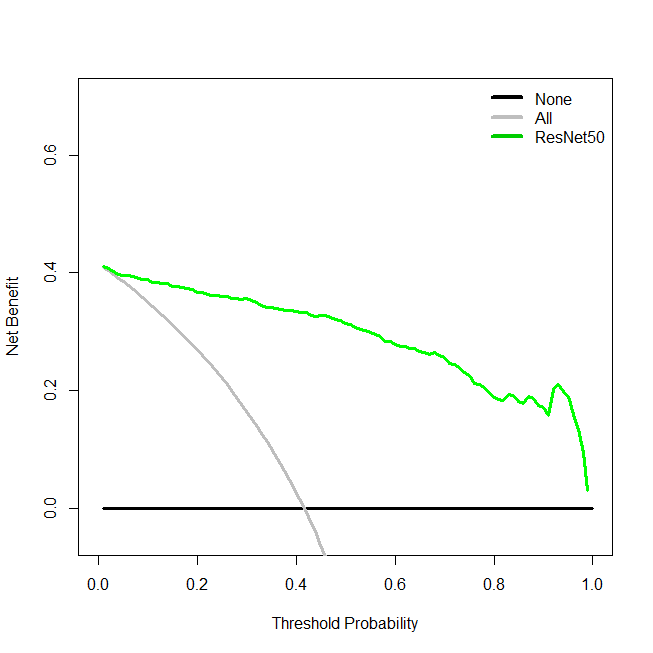


**Supplemental Figure 3.** The DCA of the training cohort indicates that when the threshold probability was above 4%, use of the deep model for predicting TACE response would gain more benefit than the “treat-all” patients or “treat-none” schemes.

**Supplementary Information** (**ResNet50)**

The deep learning model has 177 layers in total as following detailed description. We froze the weights of earlier layers (1 to 174) in the pretrained network. A series of blocks consisting of three convolutional layers (fc1000, fc1000_softmax, and classification layers_fc1000) were replaced by new layers (fc4, fc4_softmax, and classification layers_fc4). Green section indicates froze layers, and red section indicates new layers in our model.

**1 'input_1' Image Input 224x224x3 images with 'zerocenter' normalization**

**2 'conv1' Convolution 64 7x7x3 convolutions with stride [2 2] and padding [3 3 3 3]**

**3 'bn_conv1' Batch Normalization Batch normalization with 64 channels**

**4 'activation_1_relu' ReLU ReLU**

**5 'max_pooling2d_1' Max Pooling 3x3 max pooling with stride [2 2] and padding [0 0 0 0]**

**6 'res2a_branch2a' Convolution 64 1x1x64 convolutions with stride [1 1] and padding [0 0 0 0]**

**7 'bn2a_branch2a' Batch Normalization Batch normalization with 64 channels**

**8 'activation_2_relu' ReLU ReLU**

**9 'res2a_branch2b' Convolution 64 3x3x64 convolutions with stride [1 1] and padding 'same'**

**10 'bn2a_branch2b' Batch Normalization Batch normalization with 64 channels**

**11 'activation_3_relu' ReLU ReLU**

**12 'res2a_branch2c' Convolution 256 1x1x64 convolutions with stride [1 1] and padding [0 0 0 0]**

**13 'res2a_branch1' Convolution 256 1x1x64 convolutions with stride [1 1] and padding [0 0 0 0]**

**14 'bn2a_branch2c' Batch Normalization Batch normalization with 256 channels**

**15 'bn2a_branch1' Batch Normalization Batch normalization with 256 channels**

**16 'add_1' Addition Element-wise addition of 2 inputs**

**17 'activation_4_relu' ReLU ReLU**

**18 'res2b_branch2a' Convolution 64 1x1x256 convolutions with stride [1 1] and padding [0 0 0 0]**

**19 'bn2b_branch2a' Batch Normalization Batch normalization with 64 channels**

**20 'activation_5_relu' ReLU ReLU**

**21 'res2b_branch2b' Convolution 64 3x3x64 convolutions with stride [1 1] and padding 'same'**

**22 'bn2b_branch2b' Batch Normalization Batch normalization with 64 channels**

**23 'activation_6_relu' ReLU ReLU**

**24 'res2b_branch2c' Convolution 256 1x1x64 convolutions with stride [1 1] and padding [0 0 0 0]**

**25 'bn2b_branch2c' Batch Normalization Batch normalization with 256 channels**

**26 'add_2' Addition Element-wise addition of 2 inputs**

**27 'activation_7_relu' ReLU ReLU**

**28 'res2c_branch2a' Convolution 64 1x1x256 convolutions with stride [1 1] and padding [0 0 0 0]**

**29 'bn2c_branch2a' Batch Normalization Batch normalization with 64 channels**

**30 'activation_8_relu' ReLU ReLU**

**31 'res2c_branch2b' Convolution 64 3x3x64 convolutions with stride [1 1] and padding 'same'**

**32 'bn2c_branch2b' Batch Normalization Batch normalization with 64 channels**

**33 'activation_9_relu' ReLU ReLU**

**34 'res2c_branch2c' Convolution 256 1x1x64 convolutions with stride [1 1] and padding [0 0 0 0]**

**35 'bn2c_branch2c' Batch Normalization Batch normalization with 256 channels**

**36 'add_3' Addition Element-wise addition of 2 inputs**

**37 'activation_10_relu' ReLU ReLU**

**38 'res3a_branch2a' Convolution 128 1x1x256 convolutions with stride [2 2] and padding [0 0 0 0]**

**39 'bn3a_branch2a' Batch Normalization Batch normalization with 128 channels**

**40 'activation_11_relu' ReLU ReLU**

**41 'res3a_branch2b' Convolution 128 3x3x128 convolutions with stride [1 1] and padding 'same'**

**42 'bn3a_branch2b' Batch Normalization Batch normalization with 128 channels**

**43 'activation_12_relu' ReLU ReLU**

**44 'res3a_branch2c' Convolution 512 1x1x128 convolutions with stride [1 1] and padding [0 0 0 0]**

**45 'res3a_branch1' Convolution 512 1x1x256 convolutions with stride [2 2] and padding [0 0 0 0]**

**46 'bn3a_branch2c' Batch Normalization Batch normalization with 512 channels**

**47 'bn3a_branch1' Batch Normalization Batch normalization with 512 channels**

**48 'add_4' Addition Element-wise addition of 2 inputs**

**49 'activation_13_relu' ReLU ReLU**

**50 'res3b_branch2a' Convolution 128 1x1x512 convolutions with stride [1 1] and padding [0 0 0 0]**

**51 'bn3b_branch2a' Batch Normalization Batch normalization with 128 channels**

**52 'activation_14_relu' ReLU ReLU**

**53 'res3b_branch2b' Convolution 128 3x3x128 convolutions with stride [1 1] and padding 'same'**

**54 'bn3b_branch2b' Batch Normalization Batch normalization with 128 channels**

**55 'activation_15_relu' ReLU ReLU**

**56 'res3b_branch2c' Convolution 512 1x1x128 convolutions with stride [1 1] and padding [0 0 0 0]**

**57 'bn3b_branch2c' Batch Normalization Batch normalization with 512 channels**

**58 'add_5' Addition Element-wise addition of 2 inputs**

**59 'activation_16_relu' ReLU ReLU**

**60 'res3c_branch2a' Convolution 128 1x1x512 convolutions with stride [1 1] and padding [0 0 0 0]**

**61 'bn3c_branch2a' Batch Normalization Batch normalization with 128 channels**

**62 'activation_17_relu' ReLU ReLU**

**63 'res3c_branch2b' Convolution 128 3x3x128 convolutions with stride [1 1] and padding 'same'**

**64 'bn3c_branch2b' Batch Normalization Batch normalization with 128 channels**

**65 'activation_18_relu' ReLU ReLU**

**66 'res3c_branch2c' Convolution 512 1x1x128 convolutions with stride [1 1] and padding [0 0 0 0]**

**67 'bn3c_branch2c' Batch Normalization Batch normalization with 512 channels**

**68 'add_6' Addition Element-wise addition of 2 inputs**

**69 'activation_19_relu' ReLU ReLU**

**70 'res3d_branch2a' Convolution 128 1x1x512 convolutions with stride [1 1] and padding [0 0 0 0]**

**71 'bn3d_branch2a' Batch Normalization Batch normalization with 128 channels**

**72 'activation_20_relu' ReLU ReLU**

**73 'res3d_branch2b' Convolution 128 3x3x128 convolutions with stride [1 1] and padding 'same'**

**74 'bn3d_branch2b' Batch Normalization Batch normalization with 128 channels**

**75 'activation_21_relu' ReLU ReLU**

**76 'res3d_branch2c' Convolution 512 1x1x128 convolutions with stride [1 1] and padding [0 0 0 0]**

**77 'bn3d_branch2c' Batch Normalization Batch normalization with 512 channels**

**78 'add_7' Addition Element-wise addition of 2 inputs**

**79 'activation_22_relu' ReLU ReLU**

**80 'res4a_branch2a' Convolution 256 1x1x512 convolutions with stride [2 2] and padding [0 0 0 0]**

**81 'bn4a_branch2a' Batch Normalization Batch normalization with 256 channels**

**82 'activation_23_relu' ReLU ReLU**

**83 'res4a_branch2b' Convolution 256 3x3x256 convolutions with stride [1 1] and padding 'same'**

**84 'bn4a_branch2b' Batch Normalization Batch normalization with 256 channels**

**85 'activation_24_relu' ReLU ReLU**

**86 'res4a_branch2c' Convolution 1024 1x1x256 convolutions with stride [1 1] and padding [0 0 0 0]**

**87 'res4a_branch1' Convolution 1024 1x1x512 convolutions with stride [2 2] and padding [0 0 0 0]**

**88 'bn4a_branch2c' Batch Normalization Batch normalization with 1024 channels**

**89 'bn4a_branch1' Batch Normalization Batch normalization with 1024 channels**

**90 'add_8' Addition Element-wise addition of 2 inputs**

**91 'activation_25_relu' ReLU ReLU**

**92 'res4b_branch2a' Convolution 256 1x1x1024 convolutions with stride [1 1] and padding [0 0 0 0]**

**93 'bn4b_branch2a' Batch Normalization Batch normalization with 256 channels**

**94 'activation_26_relu' ReLU ReLU**

**95 'res4b_branch2b' Convolution 256 3x3x256 convolutions with stride [1 1] and padding 'same'**

**96 'bn4b_branch2b' Batch Normalization Batch normalization with 256 channels**

**97 'activation_27_relu' ReLU ReLU**

**98 'res4b_branch2c' Convolution 1024 1x1x256 convolutions with stride [1 1] and padding [0 0 0 0]**

**99 'bn4b_branch2c' Batch Normalization Batch normalization with 1024 channels**

**100 'add_9' Addition Element-wise addition of 2 inputs**

**101 'activation_28_relu' ReLU ReLU**

**102 'res4c_branch2a' Convolution 256 1x1x1024 convolutions with stride [1 1] and padding [0 0 0 0]**

**103 'bn4c_branch2a' Batch Normalization Batch normalization with 256 channels**

**104 'activation_29_relu' ReLU ReLU**

**105 'res4c_branch2b' Convolution 256 3x3x256 convolutions with stride [1 1] and padding 'same'**

**106 'bn4c_branch2b' Batch Normalization Batch normalization with 256 channels**

**107 'activation_30_relu' ReLU ReLU**

**108 'res4c_branch2c' Convolution 1024 1x1x256 convolutions with stride [1 1] and padding [0 0 0 0]**

**109 'bn4c_branch2c' Batch Normalization Batch normalization with 1024 channels**

**110 'add_10' Addition Element-wise addition of 2 inputs**

**111 'activation_31_relu' ReLU ReLU**

**112 'res4d_branch2a' Convolution 256 1x1x1024 convolutions with stride [1 1] and padding [0 0 0 0]**

**113 'bn4d_branch2a' Batch Normalization Batch normalization with 256 channels**

**114 'activation_32_relu' ReLU ReLU**

**115 'res4d_branch2b' Convolution 256 3x3x256 convolutions with stride [1 1] and padding 'same'**

**116 'bn4d_branch2b' Batch Normalization Batch normalization with 256 channels**

**117 'activation_33_relu' ReLU ReLU**

**118 'res4d_branch2c' Convolution 1024 1x1x256 convolutions with stride [1 1] and padding [0 0 0 0]**

**119 'bn4d_branch2c' Batch Normalization Batch normalization with 1024 channels**

**120 'add_11' Addition Element-wise addition of 2 inputs**

**121 'activation_34_relu' ReLU ReLU**

**122 'res4e_branch2a' Convolution 256 1x1x1024 convolutions with stride [1 1] and padding [0 0 0 0]**

**123 'bn4e_branch2a' Batch Normalization Batch normalization with 256 channels**

**124 'activation_35_relu' ReLU ReLU**

**125 'res4e_branch2b' Convolution 256 3x3x256 convolutions with stride [1 1] and padding 'same'**

**126 'bn4e_branch2b' Batch Normalization Batch normalization with 256 channels**

**127 'activation_36_relu' ReLU ReLU**

**128 'res4e_branch2c' Convolution 1024 1x1x256 convolutions with stride [1 1] and padding [0 0 0 0]**

**129 'bn4e_branch2c' Batch Normalization Batch normalization with 1024 channels**

**130 'add_12' Addition Element-wise addition of 2 inputs**

**131 'activation_37_relu' ReLU ReLU**

**132 'res4f_branch2a' Convolution 256 1x1x1024 convolutions with stride [1 1] and padding [0 0 0 0]**

**133 'bn4f_branch2a' Batch Normalization Batch normalization with 256 channels**

**134 'activation_38_relu' ReLU ReLU**

**135 'res4f_branch2b' Convolution 256 3x3x256 convolutions with stride [1 1] and padding 'same'**

**136 'bn4f_branch2b' Batch Normalization Batch normalization with 256 channels**

**137 'activation_39_relu' ReLU ReLU**

**138 'res4f_branch2c' Convolution 1024 1x1x256 convolutions with stride [1 1] and padding [0 0 0 0]**

**139 'bn4f_branch2c' Batch Normalization Batch normalization with 1024 channels**

**140 'add_13' Addition Element-wise addition of 2 inputs**

**141 'activation_40_relu' ReLU ReLU**

**142 'res5a_branch2a' Convolution 512 1x1x1024 convolutions with stride [2 2] and padding [0 0 0 0]**

**143 'bn5a_branch2a' Batch Normalization Batch normalization with 512 channels**

**144 'activation_41_relu' ReLU ReLU**

**145 'res5a_branch2b' Convolution 512 3x3x512 convolutions with stride [1 1] and padding 'same'**

**146 'bn5a_branch2b' Batch Normalization Batch normalization with 512 channels**

**147 'activation_42_relu' ReLU ReLU**

**148 'res5a_branch2c' Convolution 2048 1x1x512 convolutions with stride [1 1] and padding [0 0 0 0]**

**149 'res5a_branch1' Convolution 2048 1x1x1024 convolutions with stride [2 2] and padding [0 0 0 0]**

**150 'bn5a_branch2c' Batch Normalization Batch normalization with 2048 channels**

**151 'bn5a_branch1' Batch Normalization Batch normalization with 2048 channels**

**152 'add_14' Addition Element-wise addition of 2 inputs**

**153 'activation_43_relu' ReLU ReLU**

**154 'res5b_branch2a' Convolution 512 1x1x2048 convolutions with stride [1 1] and padding [0 0 0 0]**

**155 'bn5b_branch2a' Batch Normalization Batch normalization with 512 channels**

**156 'activation_44_relu' ReLU ReLU**

**157 'res5b_branch2b' Convolution 512 3x3x512 convolutions with stride [1 1] and padding 'same'**

**158 'bn5b_branch2b' Batch Normalization Batch normalization with 512 channels**

**159 'activation_45_relu' ReLU ReLU**

**160 'res5b_branch2c' Convolution 2048 1x1x512 convolutions with stride [1 1] and padding [0 0 0 0]**

**161 'bn5b_branch2c' Batch Normalization Batch normalization with 2048 channels**

**162 'add_15' Addition Element-wise addition of 2 inputs**

**163 'activation_46_relu' ReLU ReLU**

**164 'res5c_branch2a' Convolution 512 1x1x2048 convolutions with stride [1 1] and padding [0 0 0 0]**

**165 'bn5c_branch2a' Batch Normalization Batch normalization with 512 channels**

**166 'activation_47_relu' ReLU ReLU**

**167 'res5c_branch2b' Convolution 512 3x3x512 convolutions with stride [1 1] and padding 'same'**

**168 'bn5c_branch2b' Batch Normalization Batch normalization with 512 channels**

**169 'activation_48_relu' ReLU ReLU**

**170 'res5c_branch2c' Convolution 2048 1x1x512 convolutions with stride [1 1] and padding [0 0 0 0]**

**171 'bn5c_branch2c' Batch Normalization Batch normalization with 2048 channels**

**172 'add_16' Addition Element-wise addition of 2 inputs**

**173 'activation_49_relu' ReLU ReLU**

**174 'avg_pool' Average Pooling 7x7 average pooling with stride [7 7] and padding [0 0 0 0]**

**175 'fc4' Fully Connected 4 fully connected layer**

**176 'fc4_softmax' Softmax softmax**

**177 'ClassificationLayer_fc4' Classification Output crossentropyex with 'CR' and 3 other classes**
